# Inflammatory Markers, Metabolic Profile, and Psychoneurological Symptoms in Women with Breast Cancer: A Literature Review

**DOI:** 10.7759/cureus.19953

**Published:** 2021-11-28

**Authors:** Ahmad M Al-Bashaireh, Omar Khraisat, Eman K Alnazly, Mohannad Aldiqs

**Affiliations:** 1 Faculty of Nursing, Al-Ahliyya Amman University, Amman, JOR

**Keywords:** metabolic profile, metabolites, inflammatory markers, psychoneurological symptoms, breast cancer

## Abstract

Breast cancer is one of the most prevalent cancers in women. The improvement in breast cancer treatment has significantly increased the proportion of survival rate for women with breast cancer. Despite the advancement in breast cancer treatment, a great proportion of survivors suffer from co-occurring psychoneurological symptoms which impact their quality of life. The most frequently reported psychoneurological symptoms among women with breast cancer are depressive symptoms, anxiety, fatigue, sleep disturbances, and pain. These symptoms usually appear as a cluster. Inflammatory activation and serum metabolic alterations have been associated with the etiology of cancer and with various chronic neurocognitive disorders. However, to date, no studies considered the combined effects of inflammatory markers and metabolites in the development of psychoneurological symptoms in women with breast cancer especially those who were treated with chemotherapy. Further clarification of the relationships between the inflammatory markers, serum metabolic alterations, and psychoneurological symptoms in women with breast cancer should be pursued.

## Introduction and background

Breast cancer is one of the most prevalent cancers in women. According to the American Cancer Society (2020), it is expected that more than 276,480 women in the United States of America will be diagnosed with invasive breast cancer [1]. Most of these diagnosed women were projected to be in early stages (I and II) and to survive for more than five years due to the advancement in chemotherapy treatments [2].

The improvement in breast cancer treatment has significantly increased the proportion of survival rate for women, however, despite the advancement in treatment, a great proportion of breast cancer survivors suffer from co-occurring psychoneurological symptoms which may have an adverse impact on their quality of life [3-5]. The primary goal of the study is to determine the relationships among inflammatory markers, metabolites changes, and the development, persistence, and severity of the psychoneurological symptoms across time in breast cancer women treated with chemotherapy.

## Review

Methods

For this paper, PubMed and Web of Science were used to locate related literature. The database was searched without any prior inclusion or exclusion criteria for all peered reviewed articles published till May 31, 2021. In general, there are few studies in this field that were matched with our four descriptors (e.g. psychoneurological symptoms, breast cancer, inflammatory markers, and metabolic profile). Searching processes resulted in nine pertinent studies in the best scenario given that all the Medical Subject Headings (MeSH) terminologies related to the descriptors were used during the searching process. Due to the limited number of articles in searching of the database, we targeted references that were indexed in the two best recent articles related to this phenomenon which finally led to thirteen studies. Most of these studies were about psychoneurological symptoms in women with breast cancer with a majority of studies that enrolled women who were treated with chemotherapy. The first two authors extracted data from all peer-review articles in a standard form includes citation (authors, year), purpose, study design, sample size and characteristics, summary for selected variables (therapies, psychoneurological symptoms, inflammatory markers, and other factors), findings and/or limitations. Narrative analyses considered study design and data quality and validity.

Results and discussion

Eighteen articles were grouped into five categories. The first category includes two studies that proposed a new theoretical model that might explain this phenomenon. The second category includes four studies that investigated the psychoneurological symptoms as an isolated symptom. The third group has six studies that examined psychoneurological symptoms as a cluster of symptoms. The fourth category consists of four studies that examined the relationship between inflammatory markers and psychoneurological symptoms. Finally, the fifth category includes only two articles that described the metabolic profile in a patient with breast cancer. The findings under the five categories were described in the following sections and further information was provided in Table 1.

**Table 1:** Summary for studies of PNS in women with breast cancer. PNS: psychoneurological symptoms, NA: not applicable.

Publication (author, date)	Study Purpose	Study Design	Sample Size	Demographics: Age Stage Race	Therapy: Chemotherapy (CTX), Radiation Therapy (RTX)	PNS: Cluster (C), or Selected Symptom (SS)	Instruments measure PNS	Variables: Inflammatory Markers (IM) Metabolic Profiles (MP) Others	Major Findings
Category 1: Theoretical/Conceptual models									
(Lyon et al., 2014) [6]	To discuss new hypothesis for the biological basis of PNS through integration of inflammation and DNA repair (genetic and epigenetic) to understand symptoms development and persistence after chemotherapy treatment.	Theoretical: new theory	NA	AN	CTX	C: Persistent PNS: cognitive dysfunction, depression, anxiety, fatigue, sleep disturbances and pain	NA	IM: cytokines Other: Genetics	The epigenetic changes may involve DNA methylation or histone modifications, possibly through perturbations in the protein enhancer of zeste 2 (EZH2). Both telomerase shortening and the epigenetics changes may lead to chromosomal instability and development of the psychoneurological symptoms.
(Starkweather et al., 2013a) [7]	To discuss new conceptual model for the biological basis of PNS through integration of inflammation and genetic and epigenetic to understand symptoms development and persistence after chemotherapy treatment.	Theoretical: Conceptual Model	NA	NA	CTX	C: PNS cluster	NA	IM Other: Genetics	A proposed theoretical model for the PNS in women with breast cancer, including perceived stress, hypothalamic-pituitary adrenocortical axis dysfunction, inflammation, epigenetic, and genomic factors.
Category 2: PNS as an isolated symptom									
										(Aboalela et al., 2015) [8]	To determine if the exposure to chemotherapy, radiation, and perceived stress cause chromosomal instability and if it has a role in the development and sustainability of PNS associated with breast cancer.	Longitudinal-1 year (4 points)	71	51.3 years Stages (I to IIIA) 49 Caucasian, 22 Black African	CTX, RTX	SS: Stress	Perceived Stress Scale (PSS)	Other: Genetics	The impact of perceived stress on micronuclear/cytome frequencies was detected across all visits, with the highest levels of stress being reported at baseline. Also, the acquired micronuclear/ cytome abnormality frequencies were detected for race & tumor type.
(Wu et al., 2014) [9]	To examine the longitudinal associations between depressive symptoms and stress hormones over 12 months.	Longitudinal-1 year (4 points)	227	50.58 years Stages (II to III) 204 White, 23 Non-White 23	CTX, RTX	SS: stress, depression	Perceived Stress Scale (PSS) Epidemiological Studies Depression Scale (CES-D)	Other: Hormones: Cortisol, ACTH, epinephrine, nor-epinephrine	Depressive symptoms were inversely associated to cortisol levels but were positively correlated to rate of change in cortisol. Neither ACTH, epinephrine nor norepinephrine covaried with depressive symptoms.
(Hill et al., 2011) [10]	To examine the recurrence of depression and anxiety in breast cancer.	Longitudinal -1 year (2 points)	260	51-64 years Stage not provided Race not provided	Not provided	SS: depression & anxiety	Diagnostic and Statistical Manual of Mental Disorders (DSM) criteria (DSM MD and GAD)	None	Two-thirds with episodes of major depression and 40% with episodes of General Anxiety Disorder during the year after diagnosis were experiencing recurrence of previous disorder. Low social support predicted recurrence after controlling for previous disorder.
(Byar et al., 2006) [4]	To report the differences in fatigue, physical and psychological symptoms during chemotherapy administration over 1 year.	Longitudinal, descriptive design embedded in a pilot intervention study	25	Baseline average of 54.3 years and a range of 40-65 years Stages: I-II All Caucasian	CTX (Doxorubicin)	SS: fatigue, physical symptoms (pain, appetite, sleep disturbances, fatigue, bowel patterns, concentration, and appearance), anxiety	Piper Fatigue Scale (PFS) Symptom Experience Scale (SES) Hospital Anxiety and Depression Scale (HADS)	None	Fatigue levels were moderately intense during treatments and decreased significantly over time. Sleep disturbances and pain were the most frequent, intense, and distressing than other physical symptoms. Anxiety was highest at baseline, and depression was highest during the fourth chemotherapy treatment. Fatigue was correlated with other physical and psychological symptoms at some times during treatments and consistently following treatments.
Category 3: PNS as a cluster of symptoms									
										(Kim et al., 2014) [11]	To recognize and compare subgroups with different patterns of change in PNS clusters.	Secondary data analysis (3 points) from RCT	160	Age with mean of 54.6 years and range between 30–81 years Stages (0 to IV) 13 Non-Caucasian, 147 Caucasian	CTX, RTX	C: depressed mood & cognitive disturbance, fatigue, insomnia, and pain	Depression and confusion subscales of the (POMS-SF) General Fatigue Scale (GFS( Pittsburgh Sleep Quality Index (PSQI) One item asking about pain intensity (1-4 Likert) Consequence of symptoms was measured by the Functional Performance Inventory (FPI(	None	Five subgroups representing of PSN cluster intensity during therapy were recognized: the gradually increasing pattern subgroup (Group 1); the constantly low pattern subgroup (Group 2); the start low with dramatic increase & decrease pattern subgroup (Group 3); the constantly high pattern subgroup (Group 4); and the start high with dramatic decrease & leveling pattern subgroup (Group 5). The pattern Group 4 was observed in patients without previous cancer treatment experience, higher level of education, medicated with chemotherapy. At the second time follow –up point, clients in Group 4 had the most serious functional limitations.
(Thornton et al., 2010) [12]	To determine the association between neuroendocrine-immune models and the frequent concurrency of PNS (pain, depression, and fatigue).	Cross-sectional observational study	104	53 ±11 years Stage IV and recurrence cancer 93 Caucasian, 11 African American	CTX, RTX	C:pan, depression, & fatigue	Brief Pain Inventory (BPI) Center for Epidemiological Studies Depression Scale (CES-D) Fatigue Symptom Inventory (FSI)	Other: Hypothalamic–pituitary–adrenal axis, sympathetic nervous system	Latent variable analysis indicated neuroendocrine levels (cortisol, ACTH, epinephrine, & nor-epinephrine) predicted pain, depression and fatigue, while controlling for important disease and demographic variables.
(Liu et al., 2009) [13]	To explore the relationships between pre‐treatment cluster categories and longitudinal profiles symptoms during the course of chemotherapy.	Prospective longitudinal study (7 points)	76	Age with mean of 51.1 ± 9.1 years and a range between 34–79 years Stages I-III 55 Caucasian , 21 Non-Caucasian	CTX	C: sleep, fatigue, depression	Sleep Quality Index (PSQI) Multidimensional Fatigue Symptom Inventory—Short Form (MFSI-SF) Center of Epidemiological Studies-Depression (CES-D) scale	None	All women reported worse sleep, more fatigue and more depressive symptoms during treatment compared with baseline; however, women with a higher pre-treatment symptom cluster index sustained to experience worse symptoms during treatment compared women who began with fewer symptoms.
(So et al., 2009) [5]	To examine the symptom cluster of fatigue, pain, anxiety, and depression and its effect on the QOL of women with breast cancer that were receiving chemotherapy/ radiotherapy.	Descriptive Study	215	Age with mean of 51.65 ± 10.36 years and a range between 29-84 years Stages I-IV Chinese ethnicity	CTX, RTX	C: Fatigue, pain, anxiety, & depression	Chinese versions of the Brief Fatigue Inventory (BFI-C) Chinese version of the - Brief Pain Inventory (BPI-C) Hospital Anxiety and Depression Scale (HADS) Quality of life: the Chinese version of the Functional Assessment of Cancer Therapy for Breast Cancer (FACT-B)	None	Most participants reported mild-to-moderate levels of fatigue and pain. 21% and 36% of patients had an anxiety or depression disorder, respectively. Significant associations between 4 symptoms indicated the presence of the symptom cluster. Patients who received chemotherapy had a poorer QOL.
(Kim et al., 2008) [14]	To examine treatment-related symptom clusters and the effect of demographic/clinical variables on symptom clustering in women with breast cancer during treatment.	Secondary data analysis (4 points) from RCT	282	Age with mean of 55.21 ± 12.1 years and a range between 30-83 years Stages (0 to IV) 258 Caucasian, 24 Non-Caucasian	CTX, RTX	C: PNS cluster: depressed mood & cognitive disturbance, fatigue, insomnia, and pain Upper GI cluster: nausea, vomiting, decreased appetite	Depression and confusion subscales of the (POMS-SF) General Fatigue Scale (GFS( Pittsburgh Sleep Quality Index (PSQI) One item asking about pain intensity (1-4 Likert)	None	Two distinct clusters were identified: a psychoneurological cluster and an upper gastrointestinal cluster. Both clusters had a possibility to occur together during the treatment. Demographic and clinical variables were not significantly associated with symptom clusters.
(Bender et al., 2005) [2]	To identify and compare/symptom clusters across 3 phases of the disease.	Secondary data analysis (pooled analysis from 3 independent studies)	154 (Study 1 : 40, Study 2: 88, Study 3: 26)	Study 1: Age: 42.3 ± 5.3 years Stages I-II Race not provided Study 2: Age: 53.2 ± 6.1 years Stages: I-III Race not provided Study 3: Age: 55.2 ± 12.1 years Stage IV Race not provided	CTX, RTX	C: Anxiety, Depression, Anger, Vigor Fatigue, & Confusion	POMS	None	Three symptom clusters were identified corresponding to 3 different phases of the breast cancer experience. Each cluster was composed of symptoms related to fatigue, perceived cognitive impairment (memory problem and loss of concentration), and mood problems (anxiety & depression).
Category 4: PNS and inflammatory markers									
(Starkweather et al., 2017) [15]	To explore clusters of PNS and inflammation (levels of C-RP) over time in a cohort of women with early-stage breast cancer.	Prospective, longitudinal study (5 points)	75	Age with an average of 51.52 ± 10.34 years and a range between(23-71 years Stages: I, IIA, IIB, IIIA 53 Caucasian, 22 African American	CTX	C: cognitive, depression, anxiety, fatigue, sleep disturbance, pain, stress	CNS Vital Signs to measure cognitive Hospital Anxiety and Depression Scale (HADS) The Brief Fatigue Inventory (BFI) General Sleep Disturbance Scale (GSDS) The Brief Pain Inventory (BPI) Perceived Stress Scale (PSS)	IM: C-reactive protein (CRP)	Three clusters were identified: global cognition, affective symptoms, and cognitive efficiency. Across the time of the study, there was an inverse association between levels of C-reactive protein (CRP) and cognitive efficiency.
(Lyon et al., 2016) [16]	To assess the relationships cytokine to cognitive function over 2 years in early-stage breast cancer.	Prospective, longitudinal study (5 points)	75	Age with an average of 51.52 ± 10.34 years and a range between(23-71 years Stages: I, IIA, IIB, IIIA 53 Caucasian, 22 African American	CTX	C: cognitive	CNS Vital Signs to measure cognitive	IM: C-reactive protein (CRP) IM: Cytokines	Over time, there were associations between the patterns of cytokines and domain-specific cognitive functioning. It was found that cytokines from different classes were associated with cognitive performance and such associations were not limited to only prototypical cytokines.
(Starkweather et al., 2013b) [17]	To examine how symptom cluster subgroups distribute and associate with peripheral cytokine levels.	Secondary data analysis from cross-sectional study	128	Age with an average of 47.7 ± 7.7 and a range between 27 - 63 Stages I-II 80 Caucasian	None: assessment was done before CTX	C: Fatigue, & sleep disturbances, Depression, Pain severity & interference	Symptom Experience Survey Center Epidemiological Studies Depression Scale (CES-D) Brief Pain Inventory-Short Form	IM: cytokines	A significant difference between the high and low composite symptom score subgroups was found for interleukin IL-6 and IL-7.
(Lyon et al., 2008) [18]	To compare cytokine levels and patterns between women with breast cancer and the control group.	Cross-sectional	35 Positive biopsy, 24 negative biopsy	Positive Biopsy: Age: 48.5 ± 7.9 years Stages I-IV, and Neoadjuvant 23 Non-Hispanic White, 8 Non-Hispanic Black, 1 Hispanic, 2 Asian or Pacific Islander Negative Biopsy: Age: 49.9 ± 10.9 years Stages: not applicable 18 Non-Hispanic White, 6 Non-Hispanic Black	None: before receiving CTX	None	None	IM: cytokines	Compared with women without breast cancer, women with breast cancer had significantly higher levels of all systemic measured cytokines with an exception for granulocyte colony-stimulating factor (GCSF) and interferon-gamma (IF-γ). Three cytokines (GCSF, IL-6, and IL-17) were able to discriminate between the breast cancer and control groups
Category 5: PNS and metabolic profile									
										(Lyon et al., 2018) [19]	To assess the associations between metabolic pathway/ metabolomics (tryptophan) and the psychoneurological symptoms before and after chemotherapy.	Samples taken from prospective, longitudinal study (2 points: prior to initial CTX, and 1-2 week after the final CTX infusion )	19	Age 58.7 ± 5.2 years Stages: I, IIA, IIB, IIIA 13 White, 6 Black	CTX	C:pain, fatigue, depression	The Brief Pain Inventory (BPI) The Brief Fatigue Inventory (BFI ) Hospital Anxiety and Depression Scale (HADS)	Liquid Chromatography/ High-Resolution Mass Spectrometry targeted metabolomics from the tryptophan pathway	Levels of PNS (pain, fatigue, and depression) increased after chemotherapy. This study found symptoms of pain and fatigue were strongly associated with global and several targeted metabolites Concerning the tryptophan pathway, this study found women after chemotherapy had a higher level of pain and fatigue and a significantly higher concentration of acetyl-L-alanine, indoxyl sulfate, kynurenine levels, and kynurenine/ tryptophan.
(Louis et al., 2016) [20]	To determine whether plasma metabolic phenotype allow differentiating between breast and lung cancer.	Cross –sectional study	Breast Cancer 80 training cohort, & 60 for validation cohort Lung Cancer 55 training cohort & 90 for validation cohort	Breast Cancer Age: the average of age was 58 ± 11 years and a range between 24–86 years for training cohort, and average of age of 60 ± 12 years and a range between 40 –84 for the validation cohort Stage not provided for both groups Race not provided for both groups Lung Cancer Age: the average of age was 61 ± 10 years and a range between 43–88 years for training cohort, and average of age of 66 ± 9 years and a range between 46 83 for the validation cohort Stage not provided for both groups Race not provided for both groups	Not provided	NA	NA	The proton nuclear magnetic resonance ( 1H-NMR) Metabolic Profile (blood)	The 1H-NMR Spectroscopy technique was able to classify 99% of patients with breast cancer and 93% of patients with lung cancer based on their metabolic profile. Results were cross validated. Metabolic phenotyping of plasma permits discriminating between lung and breast cancer.

Conceptual/Theoretical Model: Psychoneurological Symptoms in Breast Cancer

Historically most of the studies in this field were designed to study this phenomenon based on the concept of dysfunction in the pathway of the hypothalamic-pituitary-adrenocortical axis (HPA). Starkweather et al. (2013a) and Lyon et al. (2014) proposed a new conceptual model to explain the variation of psychoneurological symptoms in women with breast cancer, and this model includes HPA and another three concepts: inflammation, epigenetic, and genomic factors [6,7]. In women with breast cancer, the telomerase shortening and the epigenetics changes may lead to chromosomal instability and development of the psychoneurological symptoms [6].

Nowadays this model is the cornerstone for most of the recent studies funded by the National Institute of Health (NIH) since it's incorporating constructs from the parent model "Symptom Science Model" that was developed by NIH [21]. The two primary constructs adopted in this model from the Symptom Science Model were the biomarkers identification and symptom cluster. This model includes concepts of genetic and inflammatory markers under the construct of biomarkers identification; however, it didn't consider metabolites or metabolic profiles as one of the biomarkers in its design.

Psychoneurological Distress as an Isolated Symptom in Breast Cancer

Articles under the second category had investigated the co-occurrence of psychoneurological distress as an isolated symptom in women with breast cancer. This group includes four longitudinal studies that were conducted over one year. Aboalela et al. (2015) reported that perceived stress was detected in overall patient visits, and it was associated with an impact on chromosomal stability during the treatment period with the highest level of stress being reported at the baseline of the study [8]. Wu et al. (2014) found depressive symptoms were negatively associated with cortisol level but not covaried with adrenocorticotropic hormone (ACTH), epinephrine, and norepinephrine [9].

Hill et al. (2011) reported the occurrences of depressive episodes in two-thirds and the general anxiety episodes in 40% of the patient during the first year after diagnosis [10]. Byar et al. (2006) reported results consistent with the findings in Aboalela et al. (2015) and Hill et al. (2011) studies in terms of anxiety and depression. Anxiety was the highest at baseline while depression was the highest during the fourth chemotherapy cycle. Also, Byar et al. (2006) found that pain and sleep disturbances were the most intense, frequent, and distressing symptoms over the other symptoms [4].

This category is characterized by longitudinal studies with adequate samples. Studies in this category examined psychoneurological symptoms in women with breast cancer as an isolated symptom, and such concept was reflected in its statistical analyses which were based on the techniques of univariate rather than the multivariate analysis. 

Psychoneurological Distress as Cluster of Symptoms in Breast Cancer

The third category has six studies that investigated psychoneurological distress as a cluster of symptoms. In this category, findings of these studies were ranked in the following order: (1) two descriptive cross-sectional studies, (2) one longitudinal study, and (3) finally three secondary data analysis studies that were based on randomized clinical trials.

Cross-sectional studies: So et al. (2009) reported the existence of significant correlations between four symptoms (e.g. fatigue, pain, anxiety, and depression), and they interpreted these findings as evidence supporting the presence of symptoms cluster in women with breast cancer [5]. Thornton, Andersen, and Blakely (2010) reported a similar cluster of symptoms (e.g. pain, depression, and fatigue) that was associated with latent variable indicated HPA (e.g. cortisol, ACTH, epinephrine, and norepinephrine) [12].

Longitudinal study: In 2009, Liu et al. found that breast cancer chemotherapy-treated women reported a triad cluster of a symptom (sleep, fatigue, and depression). Also, they found that women who started with a large cluster index continued to experience worse symptoms compared with women who began with a lower cluster index [13].

Secondary data analysis studies: Bender et al. (2005) identified three types of symptom clusters which were co-occurred with the three phases of breast cancer experience [2]. Each cluster was composed of symptoms related to fatigue, mood problems, and perceived cognitive impairment. Kim et al. (2008) reported two distinct clusters: a psychoneurological cluster (depressed mood, cognitive disturbance, fatigue, insomnia, and pain), and an upper gastrointestinal cluster (nausea, vomiting, and decreased appetite) [14].

Kim et al. (2008) reported that demographic and clinical variables were not significantly associated with symptom clusters [14]. Interestingly, this finding was inconsistent with the third study published by the same authors which found that a higher level of education and chemotherapy treatment were significantly associated with a higher and constant pattern of symptom clusters [11].

The third category provides support for the existence of psychoneurological distress as a cluster of symptoms in women with breast cancer. The most commonly reported symptoms in the cluster of psychoneurological symptoms were depressed mood, cognitive disturbance, fatigue, insomnia, and pain.

Psychoneurological Symptoms and Inflammatory Markers in Breast Cancer

Inflammatory markers are chemical substances released by human body cells which have an effect on the interaction and communication between cells. Lyon et al. (2008) reported that all systemic cytokines were higher in women with a positive biopsy for breast cancer compared with the women with negative biopsy results [18]. This study was followed by Starkweather et al. (2013b) who reported significant differences between high and low symptoms composite score for IL-6 and IL-7 [17].

The findings in both studies were promising and maybe help to understand the biological mechanisms underlying the development of the psychoneurological symptoms. However, there are many limitations in the two studies. First, psychoneurological symptoms were not investigated in Lyon et al. (2008) study. Second, Starkweather et al. (2013b) study was a secondary data analysis based on an original experiment that did not monitor participants over a long time. The limitations of these two studies were considered by two longitudinal published studies [15,16]. Lyon et al. (2016) found there were significant associations between cognitive performance and cytokines from different classes [16]. Moreover, Starkweather et al. 2017 found that across the time of the study there was an inverse association between levels of C-reactive protein (CRP) and cognitive efficiency [15].

Psychoneurological Symptoms and Metabolic Profile in Breast Cancer

Metabolic profile or the metabolic phenotype is a reflection of the end products of cellular processes, and the changes in its concentration either in tissue or the circulation. There are many reported evidence that cancer cell metabolism differs from that of normal cell metabolism [22]

The advances in metabolomics as a biomarker were widely used across different diseases, and it was found to be helpful in the prediction and prognosis of the illnesses. ^1^H-NMR spectroscopy is one of the noninvasive techniques that can detect more than 100 metabolites. Louis et al. (2016) found that ^1^H-NMR spectroscopy technique was able to classify 99% of patients with breast cancer based on their metabolic profile [20]. A recent pilot study correlates between global and targeted metabolic phenotypes and the psychoneurological symptoms in women with breast cancer were encouraging [19]. This study found symptoms of pain and fatigue were strongly associated with global and several targeted metabolites [19]. Concerning the tryptophan pathway, this study found women after chemotherapy had a higher level of pain and fatigue and a significantly higher concentration of acetyl-L-alanine, indoxyl sulfate, kynurenine levels, and kynurenine/tryptophan [19].

This category provides support for the existence of a correlation between psychoneurological and the metabolic phenotype. However, these studies were few and in the infancy stage. Thus more studies with various designs were needed. Further, such studies were needed to consider the combined or the simultaneous alteration in inflammatory markers and metabolite and their influence on the development, persistence, and severity of psychoneurological symptoms in women with breast cancer.

**Figure 1:**
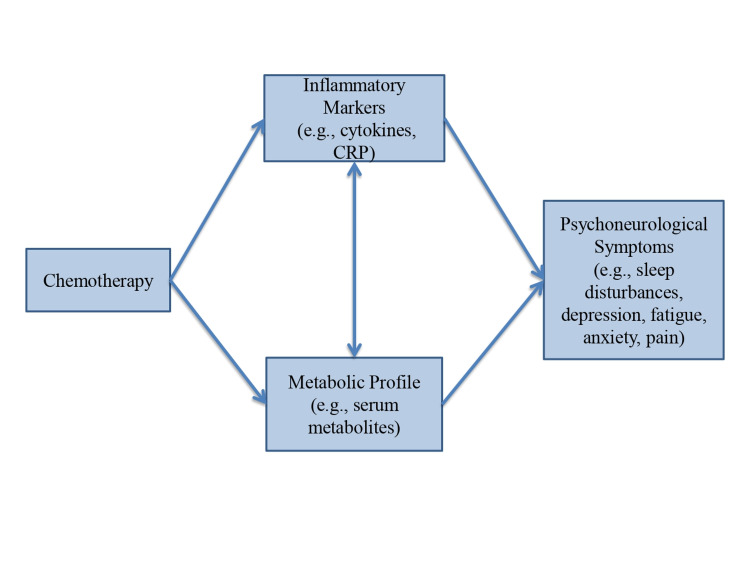
Proposed theoretical framework. CRP: C-reactive protein.

Findings of this review were summarized in a proposed model. The proposed theoretical model/framework was developed to explain the phenomenon of development, persistence, and severity of psychoneurological symptoms in women with breast cancer that was treated by chemotherapy (Figure 1).

## Conclusions

In general, several pieces of evidence support the existence of psychoneurological symptoms as clusters in women with breast cancer. However, to date, no study has considered the combined effects of inflammatory activation and metabolic profile in the development of the psychoneurological symptoms in women with breast cancer. We recommend further studies should shed light on the following phenomenon in that chemotherapy will induce an alteration in the inflammatory markers and serum metabolites among breast cancer women treated with chemotherapy, and such alterations are required for the development, persistence, and severity of the psychoneurological symptoms.

## References

[1] (2021). American Cancer Society: cancer facts and figures. https://www.cancer.org/content/dam/cancer-org/research/cancer-facts-and-statistics/annual-cancer-facts-and-figures/2020/cancer-facts-and-figures-2020.pdf..

[2] Bender CM, Ergÿn FS, Rosenzweig MQ, Cohen SM, Sereika SM (2005). Symptom clusters in breast cancer across 3 phases of the disease. Cancer Nurs.

[3] Roscoe JA, Morrow GR, Hickok JT, Bushunow P, Matteson S, Rakita D, Andrews PL (2002). Temporal interrelationships among fatigue, circadian rhythm and depression in breast cancer patients undergoing chemotherapy treatment. Support Care Cancer.

[4] Byar KL, Berger AM, Bakken SL, Cetak MA (2006). Impact of adjuvant breast cancer chemotherapy on fatigue, other symptoms, and quality of life. Oncol Nurs Forum.

[5] So WK, Marsh G, Ling WM, Leung FY, Lo JC, Yeung M, Li GK (2009). The symptom cluster of fatigue, pain, anxiety, and depression and the effect on the quality of life of women receiving treatment for breast cancer: a multicenter study. Oncol Nurs Forum.

[6] Lyon D, Elmore L, Aboalela N (2014). Potential epigenetic mechanism(s) associated with the persistence of psychoneurological symptoms in women receiving chemotherapy for breast cancer: a hypothesis. Biol Res Nurs.

[7] Starkweather AR, Lyon DE, Elswick RK Jr, Montpetit AJ, Conley Y, McCain NL (2013). A conceptual model of psychoneurological symptom cluster variation in women with breast cancer: bringing nursing research to personalized medicine. Curr Pharmacogenomics Person Med.

[8] Aboalela N, Lyon D, Elswick RK Jr, Kelly DL, Brumelle J, Bear HD, Jackson-Cook C (2015). Perceived stress levels, chemotherapy, radiation treatment and tumor characteristics are associated with a persistent increased frequency of somatic chromosomal instability in women diagnosed with breast cancer: a one year longitudinal study. PloS One.

[9] Wu SM, Yang HC, Thayer JF, Andersen BL (2014). Association of the physiological stress response with depressive symptoms in patients with breast cancer. Psychosom Med.

[10] Hill J, Holcombe C, Clark L (2011). Predictors of onset of depression and anxiety in the year after diagnosis of breast cancer. Psychol Med.

[11] Kim HJ, McDermott PA, Barsevick AM (2014). Comparison of groups with different patterns of symptom cluster intensity across the breast cancer treatment trajectory. Cancer Nurs.

[12] Thornton LM, Andersen BL, Blakely WP (2010). The pain, depression, and fatigue symptom cluster in advanced breast cancer: covariation with the hypothalamic-pituitary-adrenal axis and the sympathetic nervous system. Health Psychol.

[13] Liu L, Fiorentino L, Natarajan L (2009). Pre-treatment symptom cluster in breast cancer patients is associated with worse sleep, fatigue and depression during chemotherapy. Psychooncology.

[14] Kim HJ, Barsevick AM, Tulman L, McDermott PA (2008). Treatment-related symptom clusters in breast cancer: a secondary analysis. J Pain Symptom Manage.

[15] Starkweather A, Kelly DL, Thacker L, Wright ML, Jackson-Cook CK, Lyon DE (2017). Relationships among psychoneurological symptoms and levels of C-reactive protein over 2 years in women with early-stage breast cancer. Support Care Cancer.

[16] Lyon DE, Cohen R, Chen H (2016). Relationship of systemic cytokine concentrations to cognitive function over two years in women with early stage breast cancer. J Neuroimmunol.

[17] Starkweather AR, Lyon DE, Elswick RK Jr, Montpetit A, Conley Y, McCain NL (2013). Symptom cluster research in women with breast cancer: a comparison of three subgrouping techniques. Adv Breast Cancer Res.

[18] Lyon DE, McCain NL, Walter J, Schubert C (2008). Cytokine comparisons between women with breast cancer and women with a negative breast biopsy. Nurs Res.

[19] Lyon DE, Starkweather S, Yao Y (2018). Pilot study of metabolomics and psychoneurological symptoms in women with early stage breast cancer. Biol Res Nurs.

[20] Louis E, Adriaensens P, Guedens W (2016). Metabolic phenotyping of human blood plasma: a powerful tool to discriminate between cancer types?. Ann Oncol.

[21] Cashion AK, Grady PA (2015). The National Institutes of Health/National Institutes of Nursing Research intramural research program and the development of the National Institutes of Health Symptom Science Model. Nurs Outlook.

[22] Amoêdo ND, Valencia JP, Rodrigues MF, Galina A, Rumjanek FD (2013). How does the metabolism of tumour cells differ from that of normal cells. Biosci Rep.

